# Fibroblasts Impair Migration and Antitumor Activity of NK-92 Lymphocytes in a Melanoma-on-Chip Model

**DOI:** 10.3390/bioengineering10010052

**Published:** 2022-12-30

**Authors:** Ilenia Iaia, Virginia Brancato, David Caballero, Rui L. Reis, Massimo Aglietta, Dario Sangiolo, Subhas C. Kundu

**Affiliations:** 1 Candiolo Cancer Institute, FPO-IRCCS, 10060 Candiolo, Italy; 2 Department of Oncology, University of Turin, 10060 Candiolo, Italy; 3 ICVS/3B’s-PT Government Associate Laboratory, 3B’s Research Group, I3Bs-Research Institute on Biomaterials, Biodegradables and Biomimetics, University of Minho, Headquarters of the European Institute of Excellence on Tissue Engineering and Regenerative Medicine, AvePark, Parque de Ciência e Tecnologia, 4700-000 Braga, Portugal

**Keywords:** tumor-on-chip, Adoptive cell therapy, melanoma

## Abstract

Adoptive cell therapy in solid tumors, such as melanoma, is impaired, but little is known about the role that the fibroblasts present in the tumor microenvironment could exert. However, the mechanism at play is not well understood, partly due to the lack of relevant pre-clinical models. Three-dimensional culture and microfluidic chips are used to recapitulate the dynamic interactions among different types of cells in the tumor microenvironment in controlled and physiological settings. In this brief report, we propose a reductionist melanoma-on-a-chip model for evaluating the essential role of fibroblasts in the antitumor activity of lymphocytes. To this end, 3D melanoma spheroids were monocultured and co-cultured with human dermal fibroblasts and the NK-92 cell migration towards the tumor compartment was tested in a commercially available microfluidic device. Utilizing confocal microscopy, we observed the different recruitment of NK-92 cells in the presence and absence of fibroblasts. Our results show that fibroblasts’ presence inhibits immune effector recruiting by exploiting a 3D pre-clinical tumor model.

## 1. Introduction

Our current understanding of cancer biology results from in vitro and in vivo studies on animal models and plastic, flat culture dishes. The former have provided critical insights about the pathophysiology of the disease but are associated with severe disadvantages, in particular, the lack of the human immune system. The latter mainly rely on 2D cell cultures. Still, they are incapable of capturing all the biochemical and biophysical interactions among the several types of cells dwelling in the tumor microenvironment (TME). The advent of immunotherapy pushed a paradigm shift in cancer treatment, where it is necessary to develop new strategies for mimicking the tumor–immune system interaction in vitro and investigating the dynamic interactions between cell therapy and solid tumors. Adoptive cell therapy (ACT) is an innovative approach to fighting tumors, involving the intravenous infusion of natural or engineered autologous or allogeneic immune cells after ex vivo expansion and activation [1,2,3,4]. Despite its success and benefit in hematological malignancies [5,6], ACT faces disappointing clinical results in solid tumors [7]. The biological and molecular mechanisms underlying therapeutic success have not been fully explored. In 2D assays, tumor and immune cells are co-cultured together, threatening a realistic representation of the recruitment process of the therapeutic approach.

Moreover, immune cells suspended in a medium easily recognize cancer cells seeded in a monolayer on plastic dishes. Hence, it is evident that the killing activity of immune cells is simplified, not fully accounting for the inhibitory dynamics occurring in vivo [8]. Notably, 2D systems based only on the interaction between immune and cancer cells do not consider the endothelial network, fibroblasts’ contribution or highly dense ECM in the recognition and subsequent killing of cancer cells. The homing of antitumor lymphocytes and their interaction with the tumor in a complex microenvironment is yet to be understood. Further, the observed poor efficacy has been partially attributed to the lack of understanding of how ACT functions in a solid TME [9,10,11,12]. Fibroblasts, representing the most copious stromal cells in solid tumors, secrete cytokines and chemokines directly involved in immune escape (e.g., TGF^®^, VEGF, IL-6 and CXCL1/2) and the inhibition of ACT [13,14]. For instance, the HLA-independent activity of NK cells is inhibited by fibroblasts, suppressing the expression of NK receptors, releasing perforins and granulase B and secreting TNFα and IFNγ [14,15,16,17,18]. In recent years, many studies have focused on developing models that recapitulate a realistic TME [12,19,20,21,22]. Particularly, microfluidic technology has already demonstrated its superior ability to reproduce actual events of cancer, filling the gap between unrealistic 2D in vitro and non-predictive in vivo studies. Microfluidic devices containing multiple channels can produce cell confinement and spatial distribution. Compartmentalization is essential for studying the behavior of the recruitment of effectors, such as NK cells, toward solid tumors and their TME [23,24,25]. To this end, melanoma is chosen as the solid tumor to explore the immunotherapy potential using three-dimensional (3D) tumor models [26,27]. The NK-92 lymphocyte cell line represents a simple ACT approach to investigate the possibility of tumor-infiltrating lymphocytes as an immunotherapy approach in melanoma. 

Solid tumors are populated not only by cancer cells but also by fibroblasts or immune cells. Our main aim is to show that fibroblasts’ presence impairs ACT efficacy using a microfluidic device that can better mimic solid tumor and immune cell interactions. This brief communication describes the development of a dynamic melanoma-on-a-chip model to investigate the interplay among melanoma cells, fibroblasts and NK-92 cells as immune effectors. Our data confirm the immunosuppressive activity of fibroblasts, restraining the recruitment and cytotoxicity of NK-92 against melanoma spheroids. 

## 2. Materials and Methods

### 2.1. Microfluidic Device Features

We used a DAX-1 chip (AIM Biotech) designed in a microscope slide format (75 mm × 25 mm) that enables three experiments in parallel. The chip allows one to mimic 3D microenvironments exploiting a 3-channel design, where 2 media channels flank a 3D central gel region. The chip was chosen due to its off-the-shelf format, which is highly compatible with all polymerizable gels, including collagen, which we decided to use. It is gas permeable and controls chemical gradients across the 3D region. Moreover, it is suitable for organotypic co-cultures and compatible with any fluorescence microscope.

### 2.2. Cell Lines 

The NK-92 cell line (DSMZ) was cultured in Alpha Minimum Essential Medium (α-MEM; Sigma-Aldrich, St. Louis, MO, USA) at 2 × 10^5^ cells/mL, supplemented with 2 mM L-glutamine, 1% PenStrep (100 U/mL Penicillin and 100 μg/mL Streptomycin; Sigma-Aldrich, St. Louis, MO, USA), 12.5% fetal bovine serum (FBS; Gibco, Thermofisher, Waltham, MA, USA), 12.5% horse serum (Gibco, Thermofisher, Waltham, MA, USA) and 100 U/mL recombinant human IL-2 (Miltenyi Biotec, Bergisch Gladbach, Germany). Fresh medium with IL-2 was added as needed. Before seeding for recruitment assays, NK-92 were stained with CellTracker™ Deep Red Dye (ThermoFisher, Waltham, MA, USA) for 30 min. The NK-92 cell line was chosen since, usually, only 10% of the lymphocytes are in patient blood, so there is a technical bottleneck. Moreover, NK-92 cells can be manipulated and used to study antibody-dependent cellular cytotoxicity [28,29].

The SK-Mel-28 cell line (ATCC) was cultured in Minimum Essential Medium (MEM; Sigma-Aldrich, St. Louis, MO, USA) supplemented with 2 mM L-glutamine, 1% PenStrep (100 U/mL Penicillin and 100 μg/mL Streptomycin; Sigma-Aldrich, St. Louis, MO, USA) and 10% FBS (Gibco, Thermofisher, Waltham, MA, USA). This cell line is commonly used for toxicity studies to represent the malignant B-Raf (V600E) mutated melanoma cell line. 

The Human Dermal Fibroblast (hDFbs) primary cell line was kindly provided by Dr. Alexandra P. Marques (3B’s Research Group—University of Minho, Portugal) and cultured in α-MEM supplemented with 2 mM L-glutamine, 1% PenStrep (100 U/mL Penicillin and 100 μg/mL Streptomycin; Sigma-Aldrich, St. Louis, MO, USA) and 10% FBS (Gibco, Thermofisher, Waltham, MA, USA).

### 2.3. Three-Dimensional Spheroid Experimental Setup

**Figure 1:**
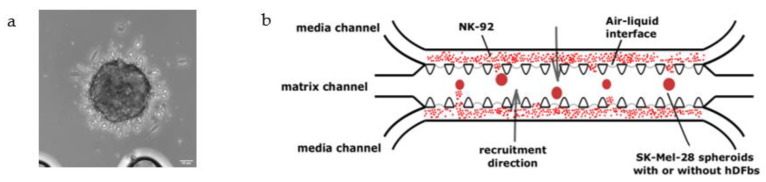
(**a**) SK-Mel-28 spheroids. Optic microscope. Magnification, 20×. Scale bar, 50 μm. (**b**) Assay setup: SK-Mel-28 preformed spheroids (in monoculture or co-culture with hDFbs) were resuspended in a collagen matrix and added into the central channel of the microfluidic chip (AIM Biotech), which had the following dimensions: length of the channel, 10.5 mm; width of the gel channel, 1.3 mm; width of the media channel, 0.5 mm; gap between posts, 0.1 mm; height of the channel, 0.25 mm. In both lateral channels, NK-92 cells labeled with a fluorescent cell tracker were resuspended in MEM medium and were added at the final concentration of 2 × 10^6^ cells/mL.

Melanoma spheroids were fabricated according to a previously published protocol (Figure 1a) [12,30]. The spheroids were formed in co-culture with hDFbs cells in a 1:2 ratio (SK-Mel-28:hDFbs). The collected melanoma spheroids (with or without hDFbs) were washed, suspended in a solution consisting of 2 mg/mL collagen rat tail type I (Gibco, Thermofisher, Waltham, MA, USA), 0.5 M NaOH and MEM 10X and were injected into the central channel of the microfluidic chip (AIM 3D Cell Culture Chip; DAX-1; AimBiotech, Singapore) (Figure 1). Next, the chip was kept in the incubator (37 °C, 5% CO_2_) for 30–40 min to promote collagen polymerization. Then, pre-labeled NK-92 cells were manually injected in the lateral channels of the chip at 2 × 10^6^ cells/mL in complete MEM medium (Figure 1b). All experiments were performed for 72 h.

### 2.4. Viability Assay and Confocal Microscopy

After 72 h, the medium with NK-92 was removed from the lateral channels, and the chip was washed with phosphate-buffered saline (PBS). A viability assay was performed by adding 1 μg/mL fluorescent calcein AM (ThermoFisher Scientific) + 2 μg/mL Propidium Iodide (PI; ThermoFisher Scientific) in PBS for 1 h and rinsed with PBS. Images were acquired with a confocal microscope (TCS SP8; Leica microsystems, Wetzlar, Germany) with a 20× oil objective. Images were processed and analyzed using Fiji software, and statistical significance was determined with unpaired *t*-tests with Welch’s correction using GraphPad Prism.

## 3. Results 

### 3.1. Recruitment Inhibition of NK-92 by Fibroblasts

**Figure 2:**
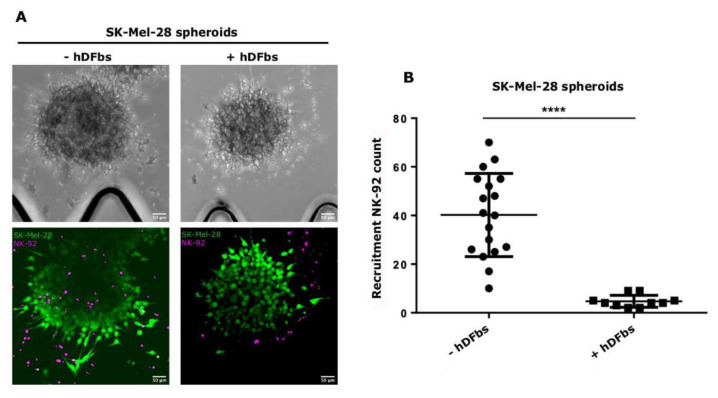
NK-92 migration: (**A**) Representative confocal images of recruitment inhibition. Magnification, 20×. Scale bar, 50 μm. On the left, monoculture of SK-Mel-28 spheroids in bright fields (on the top) and fluorescence (on the bottom; green, calcein). On the right, co-culture of SK-Mel-28 and hDFbs spheroids in bright fields (on the top) and fluorescence (on the bottom; green, calcein). In both fluorescent images, NK-92 were labeled with a fluorescent cell tracker (em. 647). (**B**) Plot shows the analysis of the recruitment inhibition of NK-92, significantly higher when hDFbs were added in the culture (“****” *p* < 0.005, unpaired t-test with Welch’s correction) (SK-Mel-28, n = 18; SK-Mel-28 + hDFbs, n = 10).

To overcome the limits associated with immune cell migration studies, we set up a microfluidic system to mimic the active migration and recruitment of NK-92 from vessels toward melanoma spheroids. The proposed system recapitulates the dynamic flow of NK-92 from the inoculation into patients and how, from vessels, they are able to reach the tumor. To this end, we decided to shed light on the role of the TME in the immune escape by adding hDFbs. Preformed SK-Mel-28:hDFbs spheroids were injected in the central channel of the microfluidic chip, and pre-labeled NK-92 were added into the lateral channels (n = 10) (see Methods). As a control, we employed SK-Mel-28 spheroids in monoculture (n = 18). After 72 h, a viability assay was performed. In the presence of fibroblasts, the recruitment of NK-92 cells toward SK-Mel-28:hDFbs spheroids was, on average, 35-fold lower than the control without fibroblasts (Figure 2). These results suggest that the presence of fibroblasts could inhibit the recruitment of NK-92 by cancer cells. 

### 3.2. Inhibition of NK-92 Anti-Melanoma Cytotoxicity by Fibroblasts 

**Figure 3:**
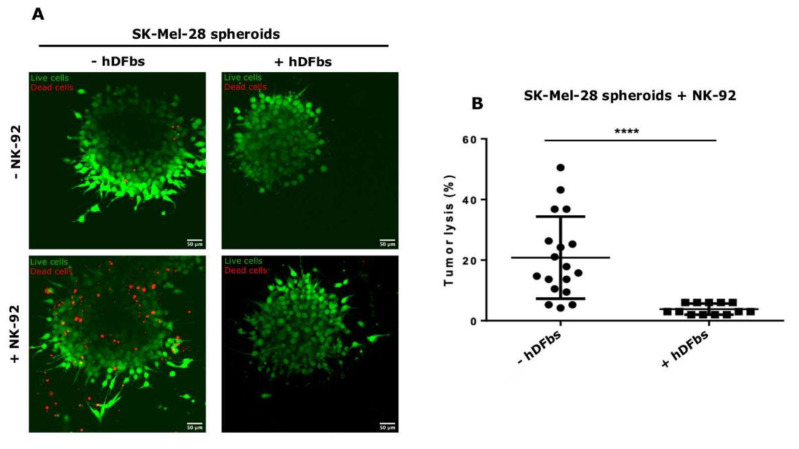
NK-92 cytotoxicity: (**A**) Representative confocal images of NK-92 cytotoxicity inhibition. Magnification, 20×. Scale bar, 50 μm. All images were acquired in fluorescence (live cells in green, em. 488, calcein; dead cells in red, em. 555, PI). On the left, monoculture of SK-Mel-28 spheroids. On the right, co-culture of SK-Mel-28 and hDFbs spheroids. In the bottom images, NK-92 were added into the culture. On the top, control experiments of spheroids without NK-92. (**B**) Plot shows the analysis of the cytotoxic inhibition of NK-92, significantly higher when hDFbs were added in the culture (“****” *p* < 0.005, unpaired *t*-test with Welch’s correction) (SK-Mel-28, n = 18; SK-Mel-28 + hDFbs, n = 13).

Given that fibroblasts inhibited the recruitment and migration of NK-92 toward the spheroids in vitro, we hypothesized that the NK-92 cytotoxic activity against solid tumor could also be hindered in vivo condition. To confirm this hypothesis, we seeded SK-Mel-28:hDFbs spheroids into the central channel, and NK-92 were added into the lateral ones (n = 18). Again, we employed SK-Mel-28 spheroids in monoculture as controls (n = 13). After 72 h, a viability assay was performed. In the presence of fibroblasts, the cytotoxic activity of NK-92 was, on average, 20% lower than that of spheroids without fibroblasts (Figure 3). This confirmed the cytotoxic inhibition activity of NK-92 by hDFbs and the high aptitude of our 3D microfluidic assay to investigate it.

## 4. Discussion and Conclusions

The immune system deeply interacts with tumor cells and the tumor microenvironment. Several solid tumors do not show relevant responses to ACT, and the mechanisms underpinning these limitations are yet to be understood. Fibroblasts represent a huge stromal component and are involved in several tumor immune escape mechanisms, confirmed by the cytotoxicity inhibition of NK-92 against melanoma spheroids. Moreover, they secrete cytokines and proteases, which may affect the interaction between immune and cancer cells. Furthermore, cytokines could also be used as costimulatory signals to promote the activity of NK cells in vivo and improve clinical response. 

However, it is still under debate which mechanisms could underpin the NK hindered by fibroblasts. Cutting NK ligands on the tumor cell surface could be one of the mechanisms involved [18]. 

Solid tumors affect effector cell penetration due to suppressive cytokines and hypoxic regions. To overcome this limitation, it is also possible to generate CAR-T cells that have a double function: targeting tumor cells and releasing cytokines that support the killing of cancer cells. Further work should be carried out to classify the cytokines derived from tumor cells and fibroblasts in terms of pro-tumor or suppressive behavior and improve the clinical relevance of this approach.

To better understand why ACT is not so efficient in solid tumors such as melanoma, it is essential to study this phenomenon in proper models. Three-dimensional systems such as microfluidic devices could embed the cancer stromal components of the tumor microenvironment involved in immune escape and cancer progression [11,17]. Microfluidic tumor models could be helpful to better investigate those mechanisms. The combination of a microfluidic device with 3D spheroids allowed us to study NK-92 interactions with melanoma spheroids in the presence and absence of fibroblasts. We confirmed a directional migration of NK-92 toward spheroids and a potent inhibition of this recruitment and cytotoxicity when fibroblasts were added. Our microfluidic assay could allow a deeper analysis of the complex architecture and interaction of ACT with solid tumors and TME to be conducted, and the analysis could be adapted to different types of tumors, therapies and stromal compounds. Behind the biological evidence of fibroblast implications in ACT response, this work bestows us with a practical and feasible 3D platform able to investigate unclarified aspects and limits of ACT in solid tumors. 

## Data Availability

Data availability is available on required.
